# Supplementary figures and images for: Polymorphisms and gene expression in the almond IGT family are not correlated to variability in growth habit in major commercial almond cultivars
Source: PLoS One. 2021 Oct 13;16(10):e0252001. doi: 10.1371/journal.pone.0252001 (PMC8513883; doi:10.1371/journal.pone.0252001)

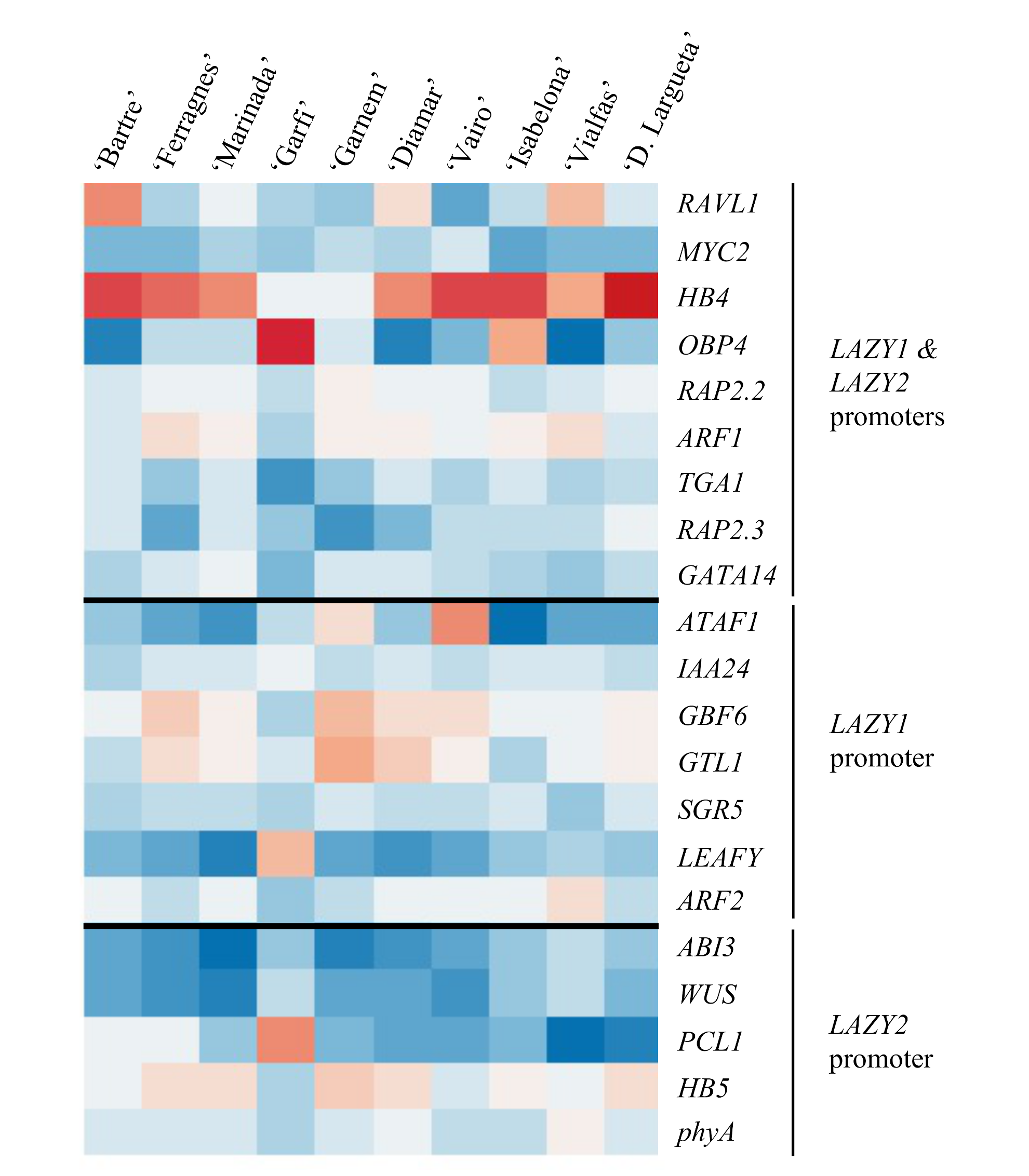

Supplement: S1 Fig — TFs are separated into three groups, whether they are expected to interact with both promoters or only one of them. Heatmap was constructed in R (https://cran.r-project.org/). (TIF) [file pone.0252001.s005.tif]
