# Supplementary material for: Polymorphisms and gene expression in the almond IGT family are not correlated to variability in growth habit in major commercial almond cultivars
Source: PLoS One. 2021 Oct 13;16(10):e0252001. doi: 10.1371/journal.pone.0252001 (PMC8513883; doi:10.1371/journal.pone.0252001)
Supplement: Attachment — Submitted filename: Response_Reviewers_Montesinosetal_revision2_AM.docx [file pone.0252001.s007.docx]

Dear Pr. Amâncio and reviewers,

We have submitted the revised version of the Manuscript “Polymorphisms and gene expression in the almond IGT family are not correlated to variability in growth habit in major commercial almond cultivars”. In the following letter and the manuscript we are addressing the point that yourself and the reviewer 3 have raised.

***1) justification of end of summer sampling;***

***2) a more robust discussion of the eventual absence of crosstalk between the expression of IGT family members and tree habit***

***Reviewer#3: Dear Prof Sara Amancio***

***I am writing my comments regarding the revised manuscript which has been submitted to PLOS ONE: Manuscript Number: PONE-D-21-15102R1.
The authors addressed all the comments by reviewers. Anyhow, I am not satisfied with their response to my comment. Still, I believe that using only one sampling time, at the end of summer to obtain the expression pattern and growth habit, is not enough to made such conclusion. However, I knew working on almond tree are not so easy to take more sampling, but I suggest that the authors add an explanation in M&M and relevant result part that why they have used end of summer to obtain the gene expression. Also, in the result part ‘Expression profiling of IGT Family members in selected almond cultivars’ fifth paragraph the sentence “it was not possible to establish a general pattern between expression levels and overall tree habit in these cultivars” should be deleted or revised since to obtain an expression pattern a greater number of samplings required.***

First, we really appreciated from reviewer 3 that he acknowledges the difficulties for sampling in almond. We choose the time point at the end of summer because it was one that was the likely to give results since the shots are in active growth but we acknowledge that we could have looked at other time points. Since we did not observed differences of expression at that point, which was following all the previous results on the gene structure and polymorphism, we technically could not invest more time and energy in a hypothesis where we have so many evidences that we will not confirm it.

However we took the opportunity that PlosOne offers the possibility to researchers to publish these kinds of “negative” results and our goal is to signal the scientific community that despite their role in other species, in almond cultivars, the IGT family genomics data and these transcriptomic data do no validate the hypothesis that they play a role in the variability observed in tree habit.

We also reviewed the discussion according to the reviewer comments on the fact that we did not observed a pattern but expression at a single time point.
